# *Salangathelphusa peractio*, a new species of lowland freshwater crab from Pulau Langkawi, Peninsular Malaysia (Crustacea, Brachyura, Gecarcinucidae)

**DOI:** 10.3897/zookeys.711.20621

**Published:** 2017-10-23

**Authors:** Peter K. L. Ng

**Affiliations:** 1 Lee Kong Chian Natural History Museum, Faculty of Science, National University of Singapore, 2 Conservatory Drive, Singapore 117377, Republic of Singapore

**Keywords:** Gecarcinucidae, Langkawi Island, Malaysia, new species, *Salangathelphusa*, taxonomy

## Abstract

A new species of lowland freshwater crab of the family Gecarcinucidae, *Salangathelphusa peractio*, is described from Langkawi, an island off the northwestern coast of peninsular Malaysia. *Salangathelphusa peractio* **sp. n.** can be separated from *S. brevicarinata* (Hilgendorf, 1882) in having a proportionately broader external orbital tooth, a distinctly concave posterolateral margin, and the terminal segment of the male first gonopod is not distinctly bent laterally outwards; and from *S. anophrys* (Kemp, 1923) by its more quadrate carapace and the terminal segment of the male first gonopod possessing a relatively longer and less curved distal part. This is sixth wholly freshwater brachyuran species known from the island.

## Introduction

The gecarcinucid genus *Salangathelphusa* was established by Bott (1968: 406) for *Parathelphusa salangensis* Ortmann, 1893, from southwestern Thailand (including Phuket) and northern Peninsular Malaysia. The choice of type species was unusual because Bott (1968) decided in the same publication that *Parathelphusa salangensis* Ortmann, 1893, described from “Salanga Island” (= Phuket) was a junior subjective synonym of *Parathelphusa brevicarinata* Hilgendorf, 1882 (misspelt by Bott [1968, 1970] as ‘*brevimarginata*’).

*Salangathelphusa* can easily be distinguished from all other southeast Asian gecarcinucids by possessing the following combination of characters: four teeth on its anterolateral margin (including the external orbital tooth); a dorsal carapace surface which is smooth with the postorbital cristae barely visible or absent; a male first gonopod which is very short and stout, with the terminal and subterminal segments clearly demarcated and a short terminal segment which has the basal part dilated and the distal part sharply tapering; and the male second gonopod has a long distal segment which is longer than half the length of the basal segment (Bott 1968, 1970; Ng 1988, 2004).

Bott (1970: 108) synonymised Paratelphusa (Paratelphusa) anophrys Kemp, 1923, with *S. brevicarinata* without any comment. Ng et al. (2008: 71), however, listed *S. anophrys* (Kemp, 1923) as a valid species of *Salangathelphusa* in their synopsis of the world Brachyura but did not elaborate. The genus *Salangathelphusa* will need to be revised as there are clearly more than the two recognised species. The author (with Darren Yeo) have examined the types of *Parathelphusa salangensis* Ortmann, 1893, and *S. anophrys* Kemp, 1923 (the type of *Parathelphusa brevicarinata* Hilgendorf, 1882, is no longer extant), as well as material from various parts of southern Thailand and northern Malaysia. It is clear that what is now called “*S. brevicarinata*” is a species complex. More material is currently being consolidated to present a more complete revision in subsequent years.

In 2015, the author examined old collections collected from the northwestern peninsular Malaysian island of Langkawi that had been collected during an expedition there by the Malayan Nature Society in 2003 (see Ahmad and Lim 2006). Among the unidentified material were two specimens of *Salangathelphusa*. Subsequent collections in Langkawi obtained more specimens of the same species, which can easily be distinguished from *S. brevicarinata* and *S. anophrys* by its distinctive male first gonopod. The present note has been prepared to make the name of this new species from Langkawi available for other studies (including conservation) because the revision of *Salangathelphusa* is still some years in the future. This present discovery of another freshwater crab species from Langkawi is somewhat surprising because the fauna of the island has been well studied. Currently four primary freshwater crab species are known – one species of Potamidae: *Stoliczia bella* Ng & Ng, 1987), and three species of Gecarcinucidae: *Sayamia sexpunctata* (Lanchester, 1906), *Phricotelphusa gracilipes* Ng & Ng, 1987, and *Siamthelphua improvisa* (Lanchester, 1902) (Ng and Ng 1987, 1989; Ng 1988, 2004). In addition, there is an endemic species of wholly freshwater sesarmid, *Geosesarma foxi* (Kemp, 1918) in the highlands of Langkawi Island (Ng 2017).

## Materials and methods

Specimens examined are deposited in the Zoological Reference Collection (**ZRC**) of the Lee Kong Chian Natural History Museum, National University of Singapore; and the Zoological Survey of India (ex Indian Museum), Calcutta, India. Measurements, in millimetres, are of the maximum carapace width and length, respectively; while the abbreviations G1 and G2 are used for the male first and second gonopods, respectively. The terminology used follows that in Ng (1988) and Davie et al. (2015).

## Systematics

### Family Gecarcinucidae Rathbun, 1904

#### Genus Salangathelphusa

Taxon classificationAnimaliaDecapodaGecarcinucidae

Bott, 1968

##### Type species.

*Parathelphusa salangensis* Ortmann, 1893, by original designation.

#### Salangathelphusa peractio sp. n.

Taxon classificationAnimaliaDecapodaGecarcinucidae

http://zoobank.org/6321FE32-9FAA-47BB-88D0-BDE7B1E6FC32

**Figure 1.**
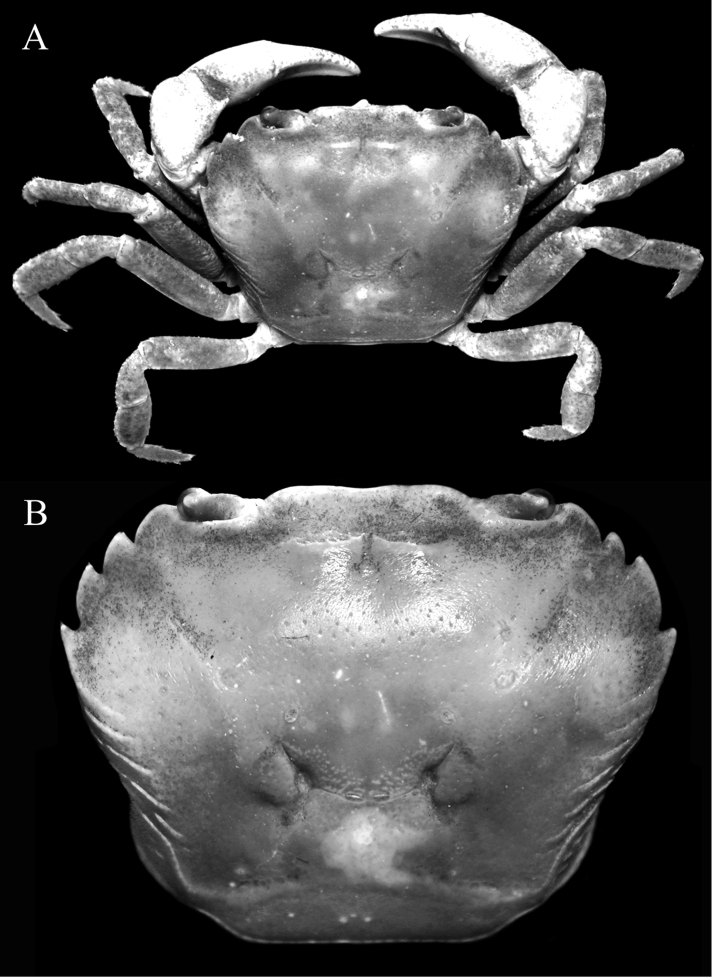
*Salangathelphusa peractio* sp. n., holotype: male (22.3 × 17.7 mm) (ZRC 2017.208), Langkawi. **A** overall habitus **B** dorsal view of carapace.

**Figure 2.**
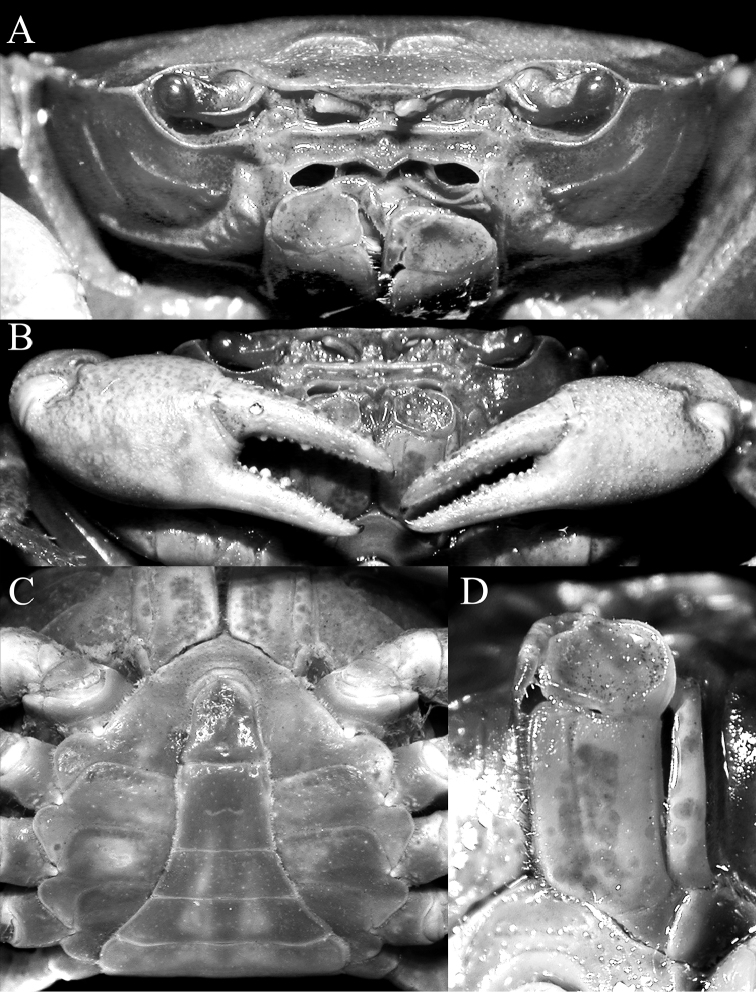
*Salangathelphusa peractio* sp. n., holotype: male (22.3 × 17.7 mm) (ZRC 2017.208), Langkawi. **A** frontal view of cephalothorax **B** outer view of chelae **C** anterior thoracic sternum and pleon **D** left third maxilliped.

**Figure 3.**
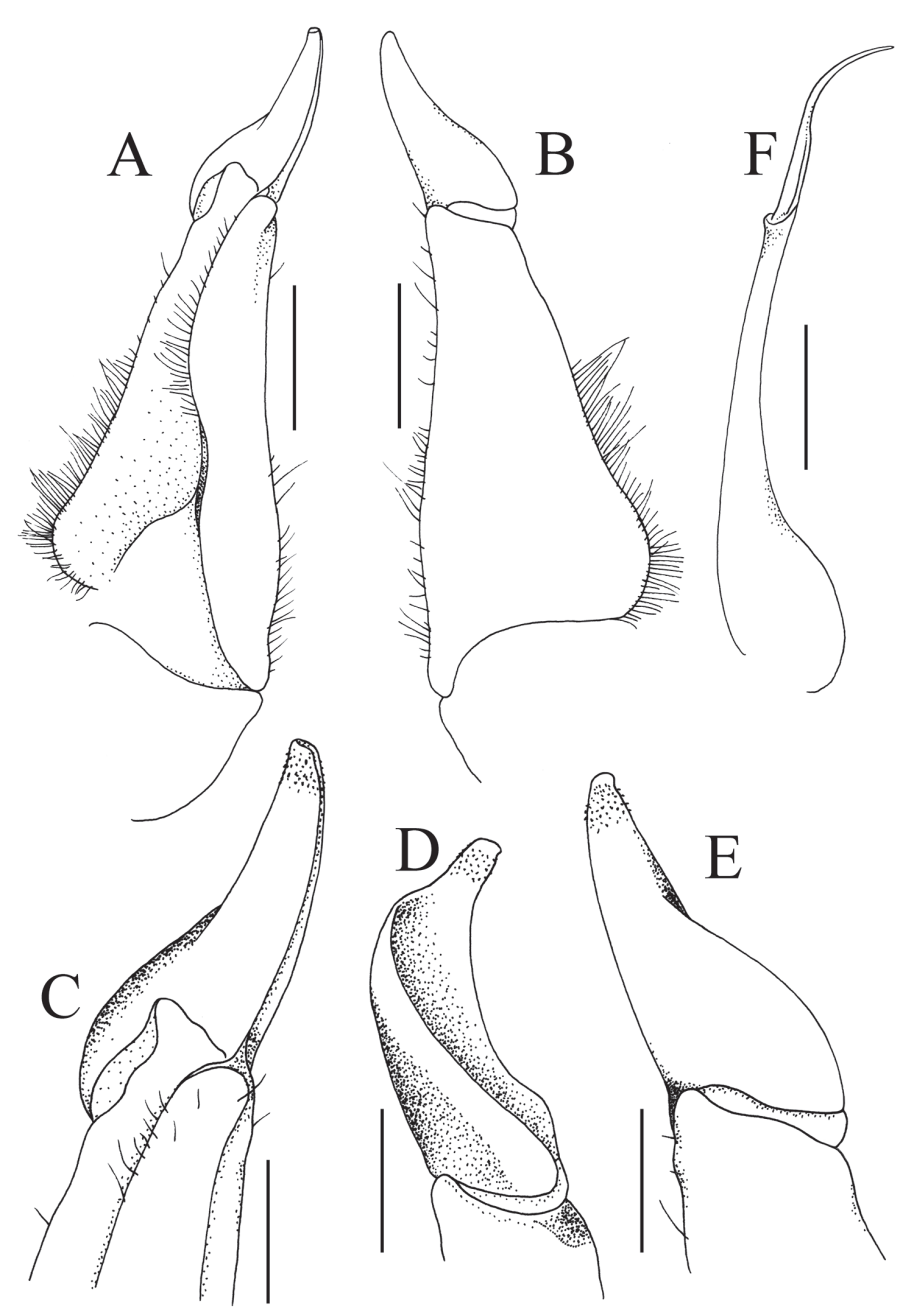
*Salangathelphusa peractio* sp. n., holotype male (22.3 × 17.7 mm) (ZRC 2017.208), Langkawi. **A** left G1 (ventral view) **B** left G1 (dorsal view) **C** distal part of left G1 (ventral view) **D** distal part of left G1 (mesial view) **E** distal part of left G1 (dorsal view) **F** left G2. Scale bars **A**, **B**, **F** 1.0 mm **C–E** 0.5 mm.

**Figure 4.**
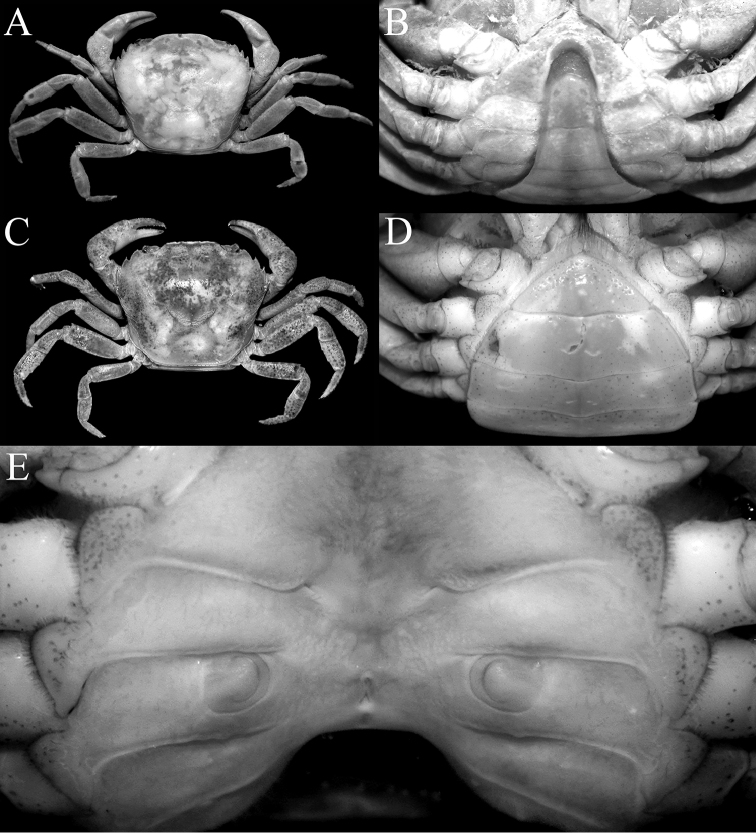
*Salangathelphusa peractio* sp. n. **A**, **B** paratype male (12.0 × 10.6 mm) (ZRC 2017.209), Langkawi **C**, **D** paratype female (21.7 × 17.3 mm) (ZRC 2017.210), Langkawi. **A**, **C** overall habitus **B** male anterior thoracic sternum and pleon **D** female anterior thoracic sternum and pleon **E** female sternopleonal cavity showing vulvae.

**Figure 5.**
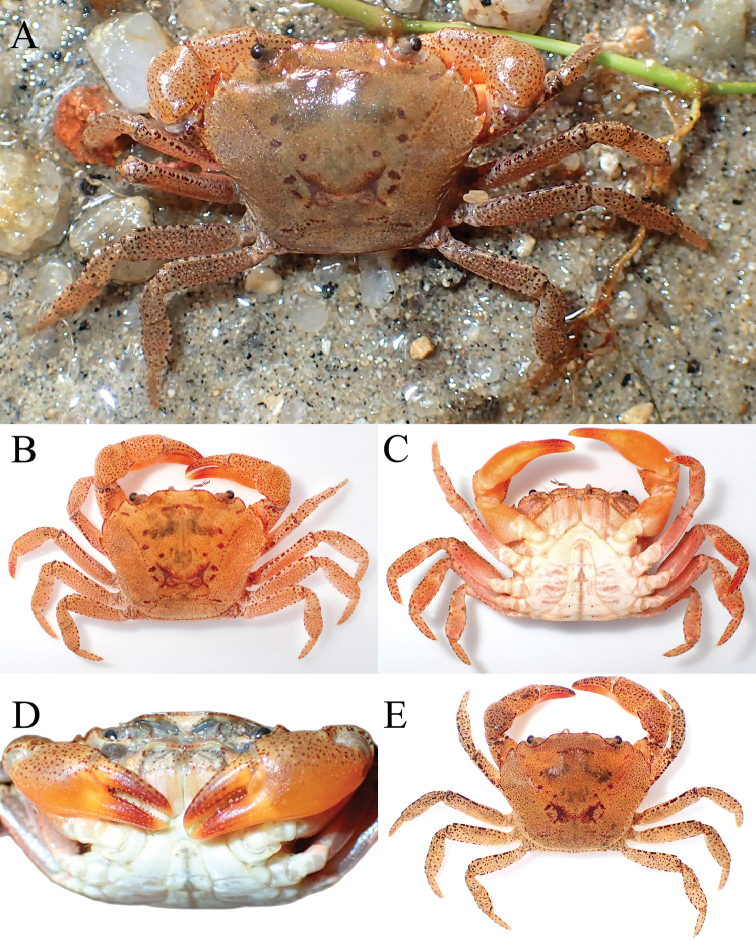
*Salangathelphusa peractio* sp. n., colour in life. **A–D** male (22.4 × 17.7 mm) (ZRC 2017.210) **E** male (21.9 × 17.3 mm) (ZRC 2017.210), Langkawi.

Figures 1 , 2 , 3 , 4 , 5

##### Material examined.

Holotype: male (22.3 × 17.7 mm) (ZRC 2017.208), Sungai Batu Asah, Kuah, Langkawi, Kedah, 6°20'22.13"N, 99°48'33.55"E, Peninsular Malaysia, coll. A. Ahmad et al., University of Sains Malaysia expedition to Langkawi, 11 April 2003. Paratypes: 1 young male (12.0 × 10.6 mm) (ZRC 2017.209), same data as holotype; 10 males (largest 22.4 × 17.7 mm, 21.9 × 17.3 mm), 2 juvenile males, 1 female (largest 21.7 × 17.3 mm), 5 young females (largest 15.3 × 13.0 mm) (ZRC 2017.210), small sandy shaded stream with rocks, 4–5 cm depth, adjacent to main river, downstream with dense waterweeds, Lubuk Semilang Park, south of Gunung Raya mountain, Langkawi, Kedah, 6°21'49.2"N, 99°47'29.39"E, Peninsular Malaysia, coll. P. K. L. Ng, 14–15 July 2017.

##### Comparative material.

*Salangathelphusa brevicarinata* (Hilgendorf, 1882): 25 males (largest 25.5 × 20.6 mm), 7 females (ZRC), Nam Tok Tone Sai, 08°01.64'N 98°21.74'E, Phuket, Thailand, coll. P. K. L. Ng and H. H. Tan, 8 April 1999; 2 males (larger 25.8 × 21.2 mm) (ZRC), same locality as above, coll. P. K. L. Ng, December 1999; 3 males, 2 females, 2 juveniles (ZRC), same locality as above, coll. S. S. C. Chong, 3 April 1985; 4 males (largest 24.7 × 19.9 mm), 2 females (ZRC), Nam Tok Kathun, 07°55.96'N, 98°19.43'E, Phuket, Thailand, coll. P. K. L. Ng and H. H. Tan, 8 April 1999. *Salangathelphusa anophrys* (Kemp, 1923): holotype male (25.4 × 19.0 mm) (ZSI C 603/1), Khao Ram, 366 m asl, Nakhon Si Thammarat mountains, Peninsular Siam (= southern Thailand), coll. M. Smith, no date; 1 male (26.9 × 20.7 mm) (ZRC 1989.2011), Sai Rung waterfall, Trang Province, southern Thailand, coll. P. Naiyanetr, 27 October 1988.

##### Diagnosis.

Carapace subquadrate, broader than long (Fig. 1); external orbital tooth broadly triangular, outer margin twice length of inner margin (Fig. 1); all ambulatory legs relatively short, merus not elongate (Fig. 1A); male pleonal somite 6 subquadrate, lateral margins gently sinuous, distal margin slightly shorter than proximal margin (Fig. 2C); posterolateral margin concave (Fig. 1); G1 with stout subterminal segment, terminal segment with tip directed upwards towards buccal cavity (Fig. 3A–E).

##### Description

**of adult male.** Carapace subquadrate, broader than long, adult carapace width to length ratio 1.25–1.27; dorsal surface gently convex, glabrous; regions poorly defined, cervical grooves shallow but, distinct, H-shaped gastrocardiac groove well developed (Fig. 1). Epigastric cristae low, sharp, smooth, separated by distinct Y-shaped groove; postorbital cristae not visible, surface smooth (Figs 1, 2A). Frontal margin gently emarginated, approximately divided into 2 broad lobes; dorsal crista of complete frontal median triangle merging with lateral cristae (Figs 1, 2A). Antennular fossae rectangular when viewed frontally, antennules folding laterally; basal antennal article quadrate, antennal flagellum short, just entering orbit (Fig. 2A). Supraorbital margin almost straight, entire; infraorbital margin gently concave, entire; orbit large, eyes completely filling orbit; sub-hepatic, pterygostomial and sub-branchial regions with low striae or smooth (Figs 1B, 2A). External orbital tooth well developed, broadly triangular, outer margin twice length of inner margin, convex, separated from first anterolateral tooth by deep triangular cleft; anterolateral margin with 3 distinct teeth, first and third of similar size, second widest, outer margins of all teeth convex with tips directed obliquely anteriorly; posterolateral margin concave, surface with strong oblique striae, distinctly converging posteriorly to almost straight posterior carapace margin (Fig. 1). Posterior margin of epistome with broadly triangular median lobe, lateral margins sinuous (Fig. 2A).

Mandibular palp 2-segmented, terminal segment prominently bilobed. Third maxilliped with ischium rectangular, with distinct longitudinal submedian sulcus; merus squarish, anterolateral margin convex, not prominently auriculiform; exopod slender, reaching to midpoint of merus, with long flagellum (Fig. 2D).

Chelipeds subequal, outer surface of merus, carpus and palm rugose; palm of right chela slightly larger; fingers not gaping, longer than palm, tips gently hooked, cutting edges without molariform teeth; merus short, stout, surface rugose with distinct subdistal tubercle on dorsal margin; carpus with strong, obliquely directed subdistal spine on inner margin; merus with low subterminal spine (Figs 1, 2B).

Ambulatory legs relatively short, stout, almost glabrous, surfaces gently rugose; second and third legs longest; merus not elongate, dorsal margin gently carinate, uneven, appearing serrated, ventral margins carinate, dorsal subdistal spine or tooth short but distinct; dactylus short, with short, sharp spines on margins (Fig. 1A).

Suture between anterior thoracic sternites 2 and 3 laterally interrupted, just visible as shallow transverse groove; sternite 3 distinctly compressed, longitudinally narrow, separated from sternite 4 by shallow lateral grooves; suture between sternites 4 and 5 medially interrupted; sutures between sternites 5/6, 6/7 and 7/8 complete with distinct median longitudinal groove on sternites 6 and 7; sternopleonal cavity extending beyond imaginary line joining anterior edge of cheliped bases, reaching to sternite 3 (Fig. 2C). Pleonal locking mechanism with strong peg-like tubercle on anterior third of sternite 5.

Pleon distinctly T-shaped, all somites and telson free; telson tongue-shaped, subequal to somite 6, lateral margins gently concave, tip broadly rounded; somite 6 subquadrate, lateral margins gently sinuous, distal margin slightly shorter than proximal margin; somites 3–5 trapezoidal (Fig. 2C).

G1 relatively short, stout; subterminal segment proportionately stout, gradually tapering towards distal half, outer margin gently sinuous; terminal segment less than half length of subterminal segment, outer margin convex, rounded tip directed upwards towards buccal cavity, inner basal part swollen, much wider than distal half, entire structure gently twisted towards sternal surface (Fig. 3A–E). G2 longer than G1; distal segment with long flagellum, ca. 0.6 times length of elongate basal segment (Fig. 3F).

##### Females.

Adult females closely resemble adult males except that the chelae are relatively more slender. The adult female pleon is ovate and covers most of the thoracic sternum (Fig. 4D), with the vulvae on the anterior half of sternite 6 being large, ovate, and possessing a prominent operculum (Fig. 4E).

##### Variation.

Smaller specimens (ca. 15 mm carapace width and below) have relatively more quadrate carapaces (width to length ratio 1.13–1.17), the merus of the ambulatory leg has a small dorsal subdistal spine and the male pleonal somite 6 is proportionately more trapezoidal in shape (Fig. 4A, B).

##### Colour.

In life, *Salangathelphusa peractio* sp. n. is light brown to orange on all its dorsal surfaces; the dorsal surface of the carapace has large reddish-brown spots and markings at or near the cervical and gastro-cardiac grooves; and the chelipeds and ambulatory legs have numerous small reddish-brown spots (Fig. 5A–C, E). The fingers of the chela are dark orange and the ventral surfaces of the cephalothorax white (Fig. 5D).

##### Etymology.

The species name is derived from the Latin word “peractio” which means “ending of a story”. It alludes to the discovery of the present freshwater species, arguably the last one the author will describe from Langkawi, ending his 30-year history with the island. Gender feminine.

##### Remarks.

**Figure 6.**
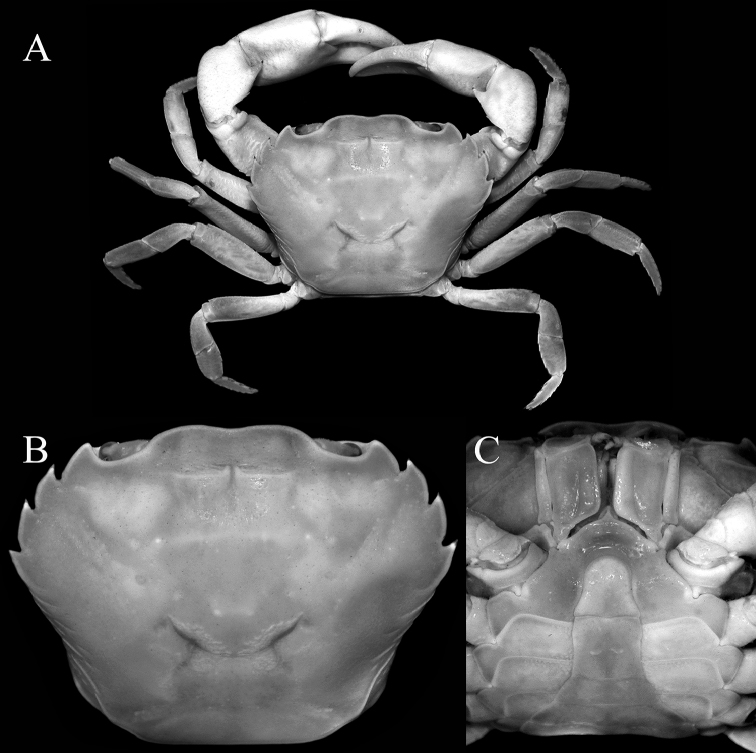
*Salangathelphusa anophrys* (Kemp, 1923), male (26.9 × 20.7 mm) (ZRC 1989.2011), Trang, Thailand. **A** overall habitus **B** dorsal view of carapace **C** male anterior thoracic sternum and pleon.

**Figure 7.**
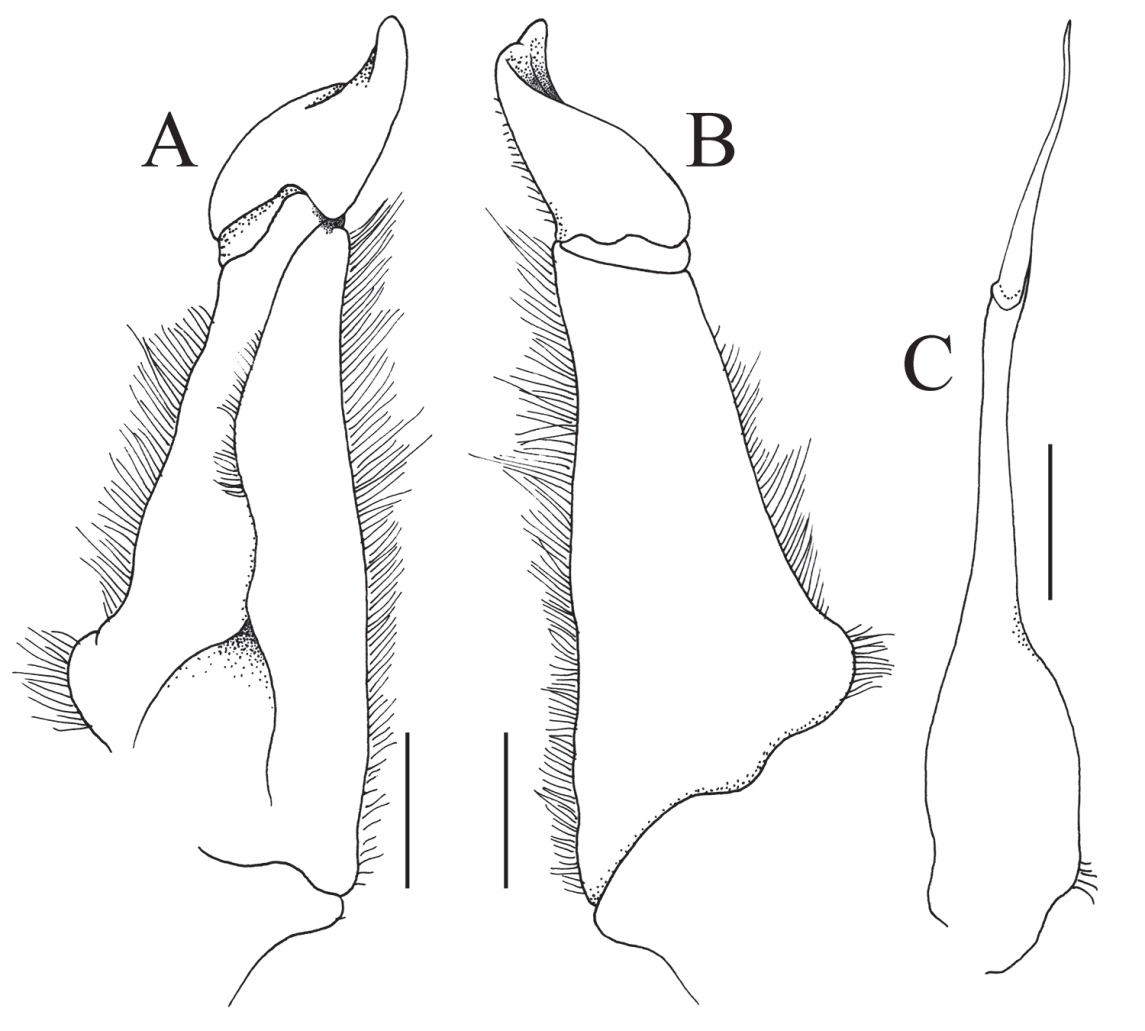
*Salangathelphusa anophrys* (Kemp, 1923), male (26.9 × 20.7 mm) (ZRC 1989.2011), Trang, Thailand. **A** left G1 (ventral view) **B** left G1 (dorsal view) **C** left G2. Scale bars 1.0 mm

*Salangathelphusa peractio* sp. n. can easily be separated from *S. brevicarinata* in that its external orbital tooth is proportionately broader (Fig. 1A, B) (external orbital tooth more acutely triangular in *S. brevicarinata*; cf. Bott 1970: pl. 20 fig. 33); the posterolateral margin is distinctly concave (Fig. 1A, B) (posterolateral margin gently concave to almost straight in *S. brevicarinata*; cf. Bott 1970: pl. 20 fig. 33); and the subterminal segment of the G1 is proportionately stouter with the distal half less slender and the terminal segment is not distinctly bent laterally outwards with the distal part directed upwards towards the buccal cavity (Fig. 3A, B, C, E) (G1 subterminal segment more slender along distal half with the terminal segment bent inwards and the distal part directed obliquely laterally in *S. brevicarinata*; cf. Bott 1970: pl. 30 fig. 78). From *S. anophrys*, *S. peractio* can be separated by its relatively more quadrate carapace (Fig. 1A, B) (carapace proportionately wider in *S. anophrys*; cf. Fig. 6A, B; Kemp 1923: pl. 4 fig. 10); and most significantly, the subterminal segment of the G1 is proportionately less stout and the terminal segment has the distal part relatively longer and less curved (Fig. 3A, B, C, E) (G1 subterminal segment stouter along entire length with the distal part of the terminal segment shorter and more strongly curved in *S. anophrys*; Fig. 7A, B).

*Salangathelphusa peractio* is known thus far only from southern streams at the base of Gunung Raya, the highest peak on Langkawi. Its distribution appears to be localised, being confined to shallow streams with fast flowing water, the substrate of the stream bed and banks being rocks of various sizes. The gecarcinucid *Siamthelphusa improvisa* was sometimes found together with *Salangathelphusa peractio*, but the former species prefers areas with dense underwater vegetation and larger rocks. At Lubuk Semilang Park, *Salangathelphusa peractio* was found only in a small area a few hundred square metres, although there are several other similar areas with similar habitats which were not accessible. The park is not a protected area and is used by the public for all manner of recreational activities which partially pollute the area as well as causing substantial disturbance to the overall habitat. How these impacts affect the crabs is not known. Unfortunately, the species is not found in any fully protected site. The restricted distribution and potential habitat impacts means that the species should perhaps be regarded as vulnerable under current conservation guidelines (see Ng and Yeo 2007; Cumberlidge et al. 2009). Its precise status will need more surveys and studies on how habitat changes will affect its population.

## Supplementary Material

XML Treatment for Salangathelphusa

XML Treatment for Salangathelphusa peractio
